# *CCT2* Mutations Evoke Leber Congenital Amaurosis due to Chaperone Complex Instability

**DOI:** 10.1038/srep33742

**Published:** 2016-09-20

**Authors:** Yuriko Minegishi, XunLun Sheng, Kazutoshi Yoshitake, Yuri Sergeev, Daisuke Iejima, Yoshio Shibagaki, Norikazu Monma, Kazuho Ikeo, Masaaki Furuno, Wenjun Zhuang, Yani Liu, Weining Rong, Seisuke Hattori, Takeshi Iwata

**Affiliations:** 1 Division of Molecular and Cellular Biology, National Institute of Sensory Organs, National Hospital Organization Tokyo Medical Center, Tokyo, Japan; 2 Ningxia Eye Hospital, Ningxia People’s Hospital, Ningxia, China; 3 Laboratory of DNA Data Analysis, National Institute of Genetics, Shizuoka, Japan; 4 National Eye Institute, National Institutes of Health, Bethesda, MD, USA; 5 Division of Biochemistry, School of Pharmaceutical Science, Kitasato University, Tokyo, Japan; 6 RIKEN Center for Life Science Technologies, Division of Genomic Technologies, Life Science Accelerator Technology Group, Transcriptome Technology Team, Yokohama, Japan

## Abstract

Leber congenital amaurosis (LCA) is a hereditary early-onset retinal dystrophy that is accompanied by severe macular degeneration. In this study, novel compound heterozygous mutations were identified as LCA-causative in *chaperonin-containing TCP-1, subunit 2 (CCT2*), a gene that encodes the molecular chaperone protein, CCTβ. The zebrafish mutants of CCTβ are known to exhibit the eye phenotype while its mutation and association with human disease have been unknown. The CCT proteins (CCT α-θ) forms ring complex for its chaperon function. The LCA mutants of CCTβ, T400P and R516H, are biochemically instable and the affinity for the adjacent subunit, CCTγ, was affected distinctly in both mutants. The patient-derived induced pluripotent stem cells (iPSCs), carrying these CCTβ mutants, were less proliferative than the control iPSCs. Decreased proliferation under *Cct2* knockdown in 661W cells was significantly rescued by wild-type CCTβ expression. However, the expression of T400P and R516H didn’t exhibit the significant effect. In mouse retina, both CCTβ and CCTγ are expressed in the retinal ganglion cells and connecting cilium of photoreceptor cells. The *Cct2* knockdown decreased its major client protein, transducing β1 (Gβ1). Here we report the novel LCA mutations in CCTβ and the impact of chaperon disability by these mutations in cellular biology.

Leber congenital amaurosis (LCA) is a hereditary early-onset congenital retinopathy that develops concomitantly with severe macular degeneration^1^. To date, nearly 20 autosomal recessive mutations have been reported to be associated with LCA (RetNet, https://sph.uth.edu/retnet/). Clinical observations of LCA patients are heterogeneous, although LCA patients are usually born almost or completely blind. Thus, the parents of LCA patients typically notice their child’s blindness within several months after birth^1^. Nearly 20% of blind school children have been diagnosed with LCA, and this disease represents a great burden on their quality of life^2^. Children with LCA are defined by a very low, or non-recordable, electroretinogram (ERG) during infancy, and the vision loss experienced by LCA patients is severe and progressive. These differences, combined with the onset of macular atrophy and pallor optic nerve head, are concomitant with the symptoms of LCA. In some cases, oculodigital signs manifest which include the rubbing or poking of a patient’s eyes by the patient. Generally, LCA is designated as an isolated retinal disorder, although certain mutations have been reported to evoke a syndromic form of LCA that involves mental retardation^3^,^4^. However, the latter observation remains controversial^5^. And the reports of the genetic cause and its pathoetiology of LCA are still limited. Here, we report the novel gene mutation that causes LCA because due to the insufficient chaperon activity.

## Results

### Compound heterozygous mutation of *CCT2* in a Chinese family with Leber Congenital Amaurosis

**Figure 1:**
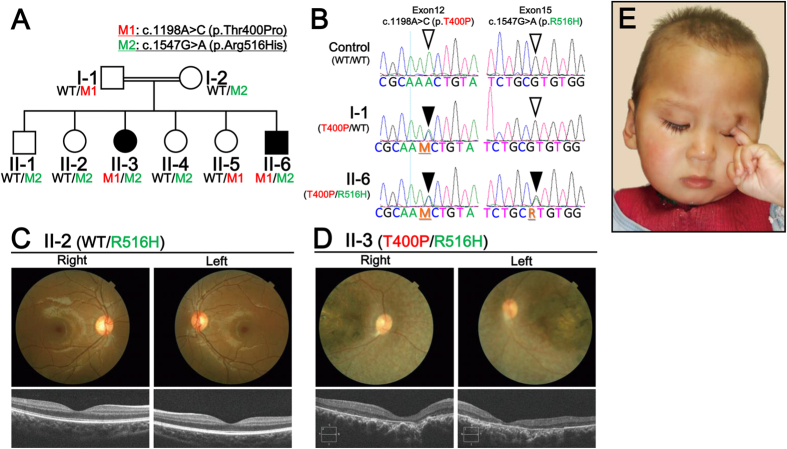
Whole Exome Sequencing (WES) and Clinical Diagnosis. (**A**) Recessive patterning of a consanguineous LCA family was performed and compound heterozygous mutations were identified. Whole exome sequencing was performed and two point mutations in *CCT2* were found, c.1198A > C and c.1547G > A, which resulted in following substitutions: p.Thr400Pro (p.T400P) and p.Arg516His (p.R516H), respectively. (**B**) *CCT2* gene mutations and corresponding amino acid substitutions in the CCTβ protein were identified. Point mutations in Exon 12 (c.1198A > C) and Exon 15 (c.1547G > A) resulted in the CCTβ missense changes, T400P and R516H, respectively. (**C**) Clinical evaluations of the non-affected individual, II-2. Upper panel: Color fundus photographs showed a normal retina. Lower Panel: An OCT examination also showed a normal retina. (**D**) Clinical evaluations of the affected individual, II-3. Upper panel: Color fundus photographs showed severe degenerative changes. The optic disc was pale and the retinal blood vessels were attenuated. A maculopathy with chorioretinal atrophy and aggregation of the pigment were also observed. Lower panel: An OCT examination showed the thinning and disorganization of the retina. Both c.1198A > C (M1 in red) and c.1547G > A (M2 in green)-carrying individuals (e.g., II-3 and II-6, respectively) exhibited retinal dystrophy and a macular degeneration phenotypes. (**E**) Facial and behavioral features of the oculodigital phenomenon (eye poking) and sunken eyes (enophthalmos) of the affected individual, II-6.

A whole exome sequencing (WES) analysis was conducted in a Chinese consanguineous family diagnosed with LCA based on the criteria mentioned above. The parents are second cousins (Supplemental Fig. 1A) and their child (II-6, in Fig. 1A) was diagnosed as blind with severe retinopathy at an age of 6 months (Supplemental Fig. 1B). All of the members of this family were examined by fundus imaging and optical coherence tomography (OCT). Six members exhibited normal fundus and OCT images (Fig. 1C), while two of the six children were further diagnosed with LCA based on the presence of attenuated blood vessels and pallor fundus images (Fig. 1D). These observations were made in combination with the habitual oculodigital signs of photophobia and eye poking (Fig. 1E), clinical hallmarks of LCA. In addition, visual acuity tests performed for the affected individuals revealed no fixation of reflection and no following of movement. Except for the retinal dystrophy, both patients have hearing disorders and language barrier. But they had no polydactyly or renal cysts, the hallmarks of Bardet-Biedl syndrome (BBS) and ciliopathy.

Further genetic analysis revealed that two novel compound heterozygous mutations, c.1198A > G and c.1547G > A, were present in this *CCT2*, and these represented unrivalled candidates in this LCA family (Fig. 1B). These mutations were located in exon 12 and exon 15 of the *CCT2* mRNA transcript, respectively.

*CCT2* encodes protein, CCTβ, one of the eight subunits of the CCT machinery^6^,^7^. The hetero-octamer ring of CCTα-θ becomes a doublet to form a cavity where nascent proteins are folded into native proteins with ATP hydrolysis^8^,^9^,^10^,^11^,^12^. While various patterns of intra-ring orientations have been reported for each subunit, CCTβ is consistently reported to be a crucial anchor in combination with CCTγ for the folding of actin and tubulin proteins in the central cavity^13^,^14^,^15^,^16^,^17^,^18^. In addition to these major components of the CCT machinery, a subset of cell division control (CDC) proteins and the recently identified key factor for retinal development, Von Hippel-Lindau disease tumor suppressor protein^19^ are also governed by the CCT machinery^20^,^21^,^22^. Together with these retinal developmental factors, myosin (*MYO7A*, synonym: *USH1B,* the cause of Usher syndrome)^23^,^24^, transducin α (*GNAT2*, the cause of Achromatopsia)^25^, and peroxisomal targeting signal 2 receptor protein (*PEX7*, the cause of Refsum disease)^26^ are also known components of the CCT machinery^27^,^28^,^29^. Recently, protein β-sheet folding by the CCT machinery was also observed in concert with the co-factor of the undergoing protein folding^30^.

Phosducin-like protein (PhLP) is a cofactor of transducin β, and dominant negative expression of PhLP specifically in photoreceptor cells has been shown to induce severe retinal degeneration in mice^31^. Thus, the CCT machinery appears to govern retinal homeostasis based on its chaperone activity. However, while an association between this machinery and genetic retinal diseases has not been identified, it is apparent that proper protein folding by CCT chaperone machinery (referred to as CCT machinery hereafter) is indeed a crucial function for retinal homeostasis. Correspondingly, alterations in a gene mutation present in one of the CCT subunits has been shown to cause retinal or neural degenerative diseases due to intracellular toxicity as a result of misfolded and aggregated proteins^32^,^33^.

Here, the LCA mutations in *CCT2* and CCTβ were found to involve the substitution of a threonine for a proline (T400P) and an arginine for a histidine (R516H), respectively. Family members that had either one of these mutations exhibited normal macula (Fig. 1C). In contrast, two of the children that carried both mutations exhibited retinal dystrophy and macular degeneration in fundus imaging, and OCT imaging revealed certain foveal hypoplasia (Fig. 1D).

### Three-dimensional Mutant Protein Structure Prediction

**Figure 2:**
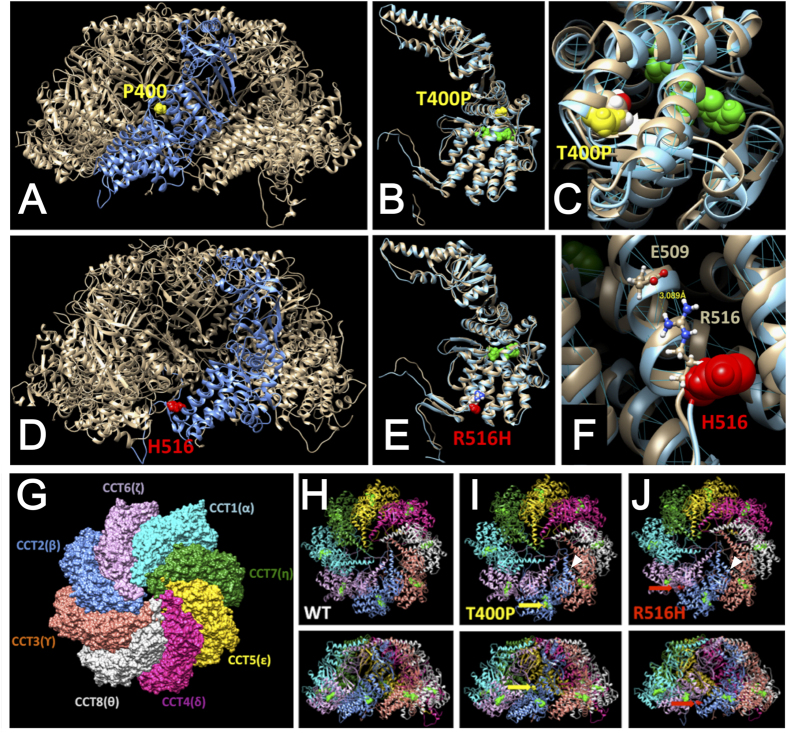
Molecular modeling for CCTβ and two CCTβ Mutant Variants. Molecular modeling of atomic structures for the CCTβ subunit, its variants, and the corresponding CCT ring complexes. Structures of the variants, T400P and R516H, are presented in Panels A–C and D–F, respectively. (**A**) Predicted structures for the CCT hetero-octamer, the T400P mutant CCTβ subunit, and the proline residue at position 400 are shown in light grey, blue, and yellow, respectively. (**B**) Structures of wild-type CCTβ (light grey) and the T400P mutant variant (light blue) are superimposed. (**C**) The T400P mutant protein structure reveals a break in the α-helix (residues 380–401) at the C-terminus. (**D**) Mutation R516H (red) is located in the inter-subunit interface of the CCT protein structure (grey). The structure for the R516H CCTβ mutant subunit is shown in blue. The histidine residue at position 516 is shown in red. (**E**) Structures of wild-type CCTβ (light grey) and the R516H mutant variant (light blue) are superimposed. (**F**) Mutation R516H at the surface of the subunit is predicted to decrease the stability of α-helix 18. An ADP molecule bound to the CCTβ subunit is green. Hydrogen bonds are blue. (**G**) Top view of the CCT hetero-octamer. (**H**) Oligomerization of wild-type CCTβ with the other CCT subunits is shown (Upper: top view, Lower: side view). (**I)** Oligomerization of the T400P variant with the other CCT subunits is shown (Upper: top view, Lower: side view). The α-helix of the T400P variant exhibited closer orientation to CCTγ than that of wild-type CCTβ as indicated with a white arrow. (**J**) Oligomerization of the R516H variant with the other CCT subunits is shown (Upper: top view, Lower: side view). The R516H variant and CCTγ are more separated than wild type CCTβ and CCTγ (indicated with a white arrow head). The T400P and R516H mutants are predicted to affect the intra-ring structure of the CCT complex.

To predict whether these mutations in CCTβ directly impact disease onset, molecular modeling of the hetero-octamer CCT machinery was conducted. Possible structural consequences of T400P and R516H are illustrated in Fig. 2, panels A–C and D–F, respectively. The change to a proline residue in position 400 appears to disrupt the α-helical conformation and destabilize several hydrogen bonds in the region of the C-cap of helix 14 (Fig. 2C). In addition, the introduction of a relatively bulky proline residue at the interface between the α-helices has the potential to cause outward movement of the surrounding helices, thereby reducing the ADP nucleotide (green) binding maintained by these helices. The other mutant, R516H, is located in the C-cap of α-helix 18. In the CCTβ subunit a positively charged arginine residue (R516) has a potential to stabilize α-helix 18 by forming a salt bridge with the negatively charged glutamic acid, E509 (Fig. 2F). Mutation of arginine R516 to histidine interrupts the salt bridge and results in the loosened α-helix structure. It is known that intermediate α-helices carrying these mutations are important for CCT intra-ring formation^6^. Furthermore, computational modeling of the CCT hetero-oligomer indicated that CCTβ mutations could affect the functional activity of the CCT machinery (Fig. 2G–J).

### CCTβ is decreased in LCA patient-derived T cells and iPSCs

**Figure 3:**
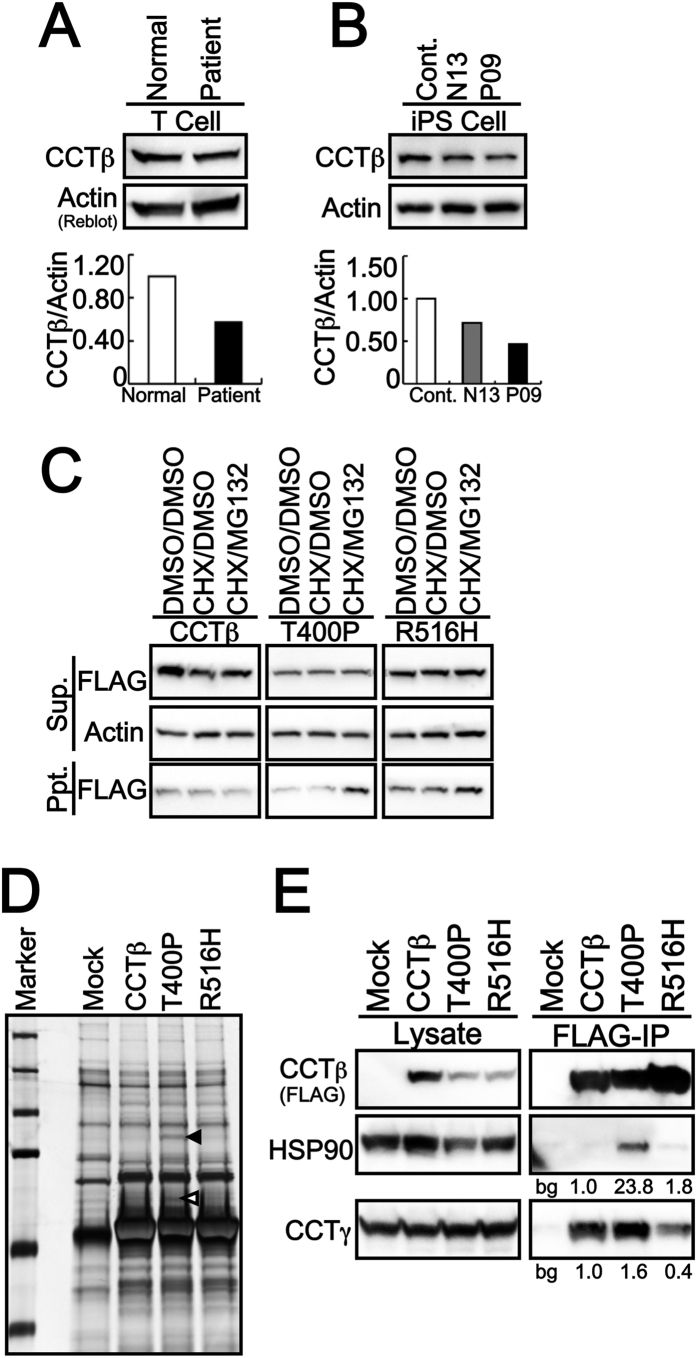
Intracellular Dynamics of CCTβ and its LCA-causing mutants. Expression levels of CCTβ protein in patient-derived T cells (**A**) and iPSCs (**B**) were analyzed by WB. Lower levels of CCTβ were detected in patient-derived T cells and iPSCs (P09) compared with the parental control iPSCs (N13) and wild-type CCTβ-carrying control iPCSs (Cont.). (**C**) Intracellular protein stability was monitored using the proteasomal degradation inhibitor, MG132, and protein synthesis was abolished with cychloheximide (CHX) treatment for 3 h. Cytosolic protein levels of T400P were lower than R516H and wild-type CCTβ. The insolubility of T400P and R516H is predicted to contribute to the degradation observed. Sup., supernatant fraction; Ppt., Precipitated fraction. (**D**) Each FLAG-tagged transfected sample was immunoprecipitated (IP) and subjected to WB and silver staining. Two distinct binding bands were observed for the T400P variant (indicated with a black arrowhead and the white arrowhead respectively). These two bands were digested with trypsin by an in-gel method and was subsequently identified as containing HSP90 (black arrowhead) and CCTγ (white arrowhead) by LC/MS/MS. (**E**) Consistent with the MS analysis data, T400P exhibited a higher affinity for HSP90 and CCTγ in IP and WB assays. In contrast, R516H exhibited a lower affinity for CCTγ. Thus, these CCTβ variants appear to affect CCTγ during oligomerization of the CCT-chaperonin ring. The number labels in (**E**) indicate the fold-change in expression compared to wild-type HSP90 or wild-type CCTβ. Bg: background from mock (empty vector transfected) control.

Computational structural predictions of T400P and R516H CCTβ identified potential defects in the oligomerization of CCT proteins. Initially, it was investigated whether these defects affected intracellular levels of CCTβ in patient-derived induced pluripotent stem cells (iPSCs). Two sets of iPSCs were established, one from an LCA patient (II-6 in Fig. 1A) and one from an unaffected parent as a parental control (I-1 in Fig. 1A). As shown in Fig. 3A,B, the levels of CCTβ protein were lower in the patient-derived T cells and iPSCs compared to the corresponding parental cells (Fig. 3A,B).

### Decreased mutant proteins and its degradation by proteasomal pathway

To elucidate the cause of the decrease in CCTβ levels, human wild-type CCTβ, and the T400P and R516H forms of CCTβ, were transiently overexpressed in HEK293T cells treated with or without the proteasomal degradation inhibitor, MG132. After cycloheximide was added to stop nascent protein synthesis, expression of T400P was found to be reduced in the soluble (supernatant) fraction, as well as in the insoluble (precipitated) fraction (Fig. 3C). The presence of T400P CCTβ in the insoluble fraction in the presence of MG132 suggests that T400P is rapidly degraded following protein synthesis. In contrast, expression of R516H CCTβ in the soluble fraction was similar to that of wild-type CCTβ, while excess R516H protein was detected in the insoluble fraction without MG132 treatment. MG132 treatment further increased the amount of R516H in the insoluble fraction, thereby suggesting that these mutant proteins undergo intracellular degradation. Taken together, these results suggest that the levels of mutant proteins in the cytosol are suppressed by intracellular degradation, presumably because of their instable structures. The observation that lower levels of mutant T400P were detected compared with mutant R516H also indicates greater instability of the T400P structure, and this may affect the cellular function of this protein.

### Distinct affinity for CCTγ of T400P and R516H Mutants

Based on the observation that the intracellular dynamics of the CCTβ mutants differed from those of wild-type CCTβ, liquid chromatography (LC)/mass spectrometry (MS)/MS proteomics was employed to examine whether this has any influence on formation of the CCTβ-associating complex^34^. A few T400P-specific bands were detected, yet there were no R516H-specific bands identified by silver staining. Two of the T400P-specific bands were identified by in-gel digestion and LC/MS/MS (Fig. 3D). The T400P form of CCTβ also exhibited greater affinity for HSP90 (indicated with a closed arrowhead in Fig. 3D), for one of the other major chaperone molecules, and for CCTγ (indicated with an open arrowhead in Fig. 3D), an adjacent subunit to CCTβ in the CCT hetero-octamer intra-ring structure. The association of each of the mutant proteins with HSP90 or CCTγ was confirmed with IP and Western blotting (WB) assays. Consistent with the results or LC/MS/MS, the HSP90 was precipitated only with T400P mutant (Fig. 3E). The CCTγ was precipitated with wild type CCTβ while the interaction seemed more abundant with T400P mutant (Fig. 3E). Unexpectedly, the R516H mutant precipitated less CCTγ compared to the wild type CCTβ or T400P mutant (Fig. 3E). Taken together, these results indicate that the expected from molecular modeling impact on CCT oligomerization is due to changes in affinity of the CCTβ-CCTγ interface.

### LCA patient-derived iPSCs are less proliferative compared to control iPSCs

**Figure 4:**
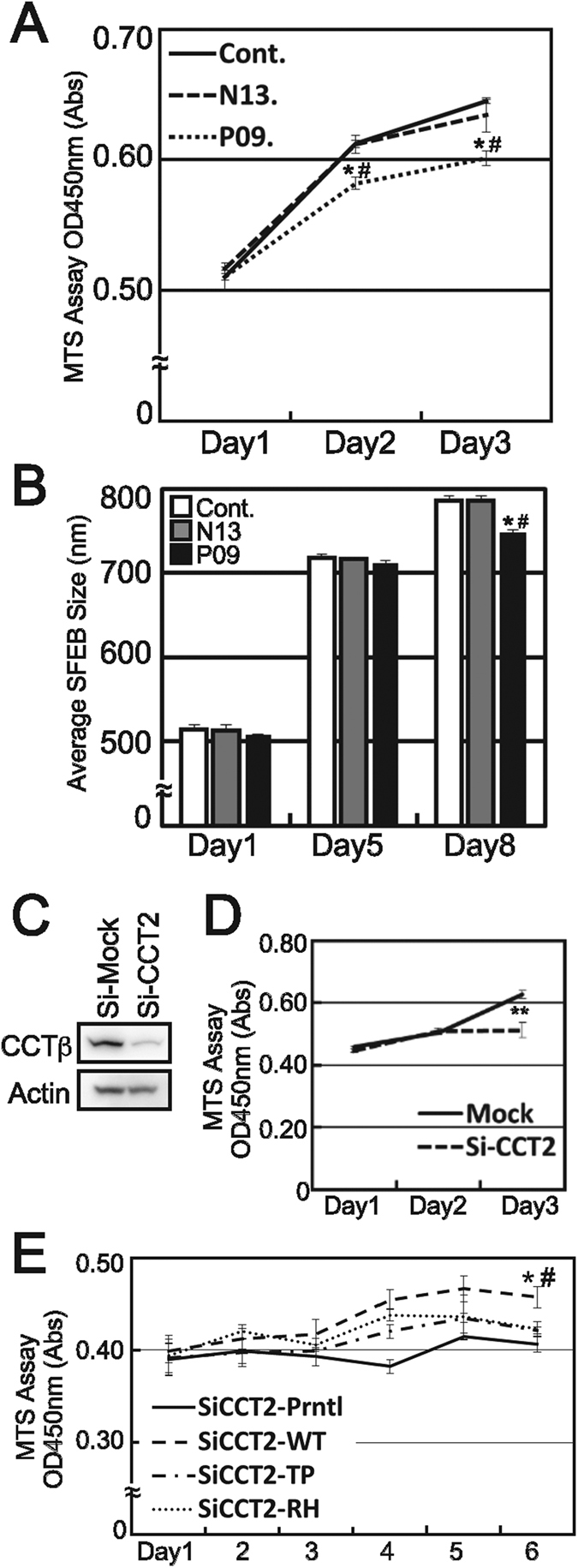
Patient-derived T400P/R516H-carrying cells are less proliferative, and these LCA-causing CCTβ mutants were failed to rescue the phenotype. (**A**) The proliferation rate for patient-derived iPSCs (P09) was significantly lower than the proliferation rate of the parental control iPSCs (N13) and the wild type CCTβ-carrying control iPCSs (Cont.) that were maintained under normal iPS culturing conditions (* and #, respectively, Student’s *t*-test, *p* < 0.05). (**B**) Growth rates according to spheroid size were also significantly smaller for patient-derived iPSCs grown in serum-free embryoid body cultures for 8 days compared to the controls (* and #, respectively, Student’s *t*-test, *p* < 0.05). (**C**) The mouse-specific knockdown of *CctT2* by siRNA significantly decreased the amount of CCTβ expressed in 661W cells (**Student’s *t*-test, p < 0.01). (**D**) Proliferation of the 661W cells was largely suppressed after 3 days of *Cct2* knockdown. (**E**) To elucidate the impact of the T400P and R516H mutants, stable 661W clones stably expressing human CCTβ, T400P, and R516H were established by lenti-virus infection. Both stable and parental 661W cell lines were transfected with *Cct2*-targeted siRNA, and cell proliferation was monitored for 6 days. Consistent with the former results, parental 661W cells and T400P-expressing 661W cells exhibited a marked decrease in cell proliferation following *Cct2* knockdown (* and #, respectively, Student’s *t*-test, *p* < 0.05). In addition, both T400P- and R516H-expressing 661W cells exhibited no statistically significant rescue effect, yet did exhibit slightly enhanced cell proliferation.

During this study, we noticed that one week later from the iPSC generation, the number of patient-derived T cells reached only 63% of the number of parental T cells present (5.4 × 10^4^ cells/ml vs. 9.5 × 10^5^ cells/ml, respectively). To explore the impact of the CCTβ mutants on cellular physiology, the cell proliferations were monitored after iPSCs establishment. A significant reduction in patient-derived iPSC growth was observed, with patient-derived T400P and R516H cells being less proliferative than the parental control (CCTβ/T400P) or normal control (CCTβ/CCTβ) cells. These results were consistent with the previously reported smaller eye phenotype observed in several zebrafish lines treated with morpholinos and mutagen-exposed zebrafish lines indicated a possibility that proliferation defects were mediated by CCTβ and CCTγ mutations^35^,^36^. Taken together, these data suggest that proliferation activity is one of the phenotypes that characterize mutant CCTβ proteins. Indeed, the patient-derived iPSCs examined in the present study exhibited a slower growth rate in 96-well MTS assays after 3 days in culture (Fig. 4A). Furthermore, the growth defect became obvious in long-term serum-free embryoid body cultures with the formation of spheroids (Fig. 4B).

### Stable expression of LCA mutants of CCTβ are lacking the rescue effects on proliferation

To investigate the impact of the CCTβ mutants on cellular growth, endogenous levels of *Cct2* were first knocked down using silencing RNA (siRNA) in the mouse photoreceptor-derived cell line, 661W (Fig. 4C). Then, stable 661W cell lines overexpressing human wild-type CCTβ, T400P CCTβ, and R516H CCTβ were generated using a lentivirus system. A mouse *Cct2*-specific siRNA sequence that did not exhibit off-target effects on human *CCT2* was used to knockdown endogenous levels of *Cct2* in the parental 661W cells, and in the three overexpression cell lines. Cell growth for each cell line was monitored for 6 d. Proliferation of the parental 661W cells was significantly decreased (shown as * in Fig. 4E), while the cells expressing wild-type CCTβ exhibited a significant rescue effect compared to the parental cells (* and # respectively, Student’s *t*-test, *p* < 0.05). In contrast, the T400P- and R516H-expressing 661W cells exhibited no statistically significant rescue effect, yet a slightly positive effect on cell proliferation was observed. These results indicate that the mutants were less effective in inducing cell proliferation than wild-type CCTβ. For mutants T400P and R516H, the former was significantly less effective than wild-type CCTβ, and there was no significant difference between R516H and wild-type CCTβ cells. These results are consistent with the structural predictions and degradation assay results described above thereby indicating that T400P CCTβ has a greater effect on the functional activity of the CCT machinery. These results also suggest that compound heterozygous mutations in *CCT2* induce a partial, and not complete, functional defect in the CCT machinery.

### CCTβ and CCTγ colocalize in photoreceptors and Retinal Ganglion Cells

**Figure 5:**
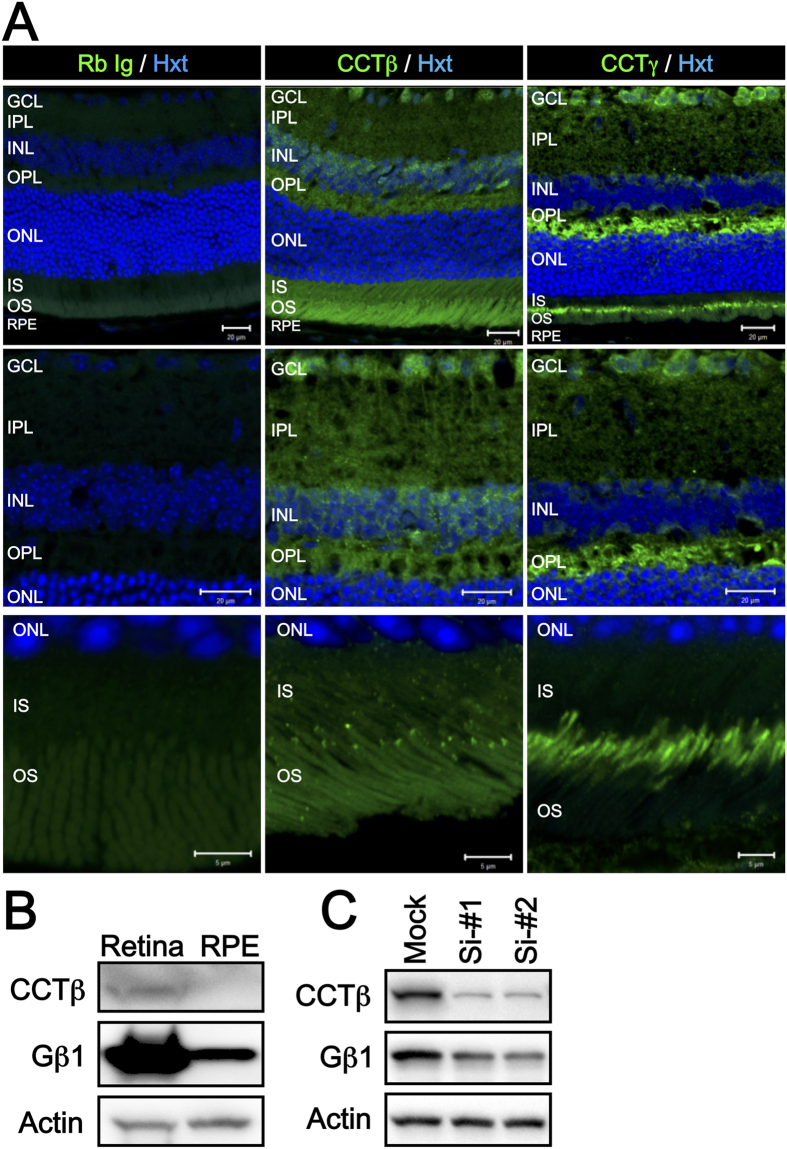
Localization of CCTβ and CCTγ in RGCs and Photoreceptor cells in Mouse Retina. (**A**) Low magnification micrographs (top, scale bar = 20 μm) show the localization of CCTβ and CCTγ in ganglion cells and photoreceptor cells, respectively. Expression of CCTβ was more dominant in the cells of ganglion cell layer (middle micrographs, scale bar = 20 μm), while both CCTβ and CCTγ strongly localized between the IS and OS (bottom micrographs, scale bar = 5 μm). In the photoreceptor cells, CCTβ was diffusely distributed in the cytosol, and was also densely localized in punctate regions between the IS and OS, similar to the basal body dominant manner in which CCTγ localized to the basal body, thereby further connecting in a cilium manner in photoreceptor cells. (**B**) Expression levels of CCTβ and Gβ1 were higher in the retinal lysates than in the RPE lysates in the mouse eye. Detection of actin was used as a loading control. (**C**) CCTβ knockdown resulted in lower levels of Gβ1 expression after 4 days of siRNA treatment in 661W cells. GCL: ganglion cell layer; IPL: inner plexiform layer; INL: inner nuclear layer; OPL: outer plexiform layer; ONL: outer nuclear layer; IS: inner segment; OS: outer segment.

While it is reasonable that the CCTβ mutations suppress cell proliferation, and this can lead to hypoplasia of the retina and the manifestation of retinal dystrophy in LCA patients, it does not explain the progression of LCA to macular degeneration. To clarify this, we next examined the tissue localization and expression of CCTβ and CCTγ, as well as their major target protein, Gβ1 (also known as transducin β in photoreceptor cells), a second messenger of photo transduction signaling. As shown in Fig. 5A, CCTβ was found to be dominantly expressed in the cells in retinal ganglion cell layer (GCL), and also expressed in photoreceptor cells. In the latter, CCTβ was diffusely localized in the cytosol, while also being localized to some dense dots just between the inner segment (IS) and the outer segment (OS). These results suggest that CCTβ localizes to the basal body or the base of the connecting cilium. CCTγ was also expressed in the RGCs, and more abundantly, in the photoreceptor cells. Of particular note is the observation that CCTγ localized just between the IS and OS, similar to CCTβ, while its localization manner was more similar to that of the connecting cilium or axonem structure. Gβ1 protein expression was also abundant in the neural retina (Fig. 5B), and siRNA-mediated knockdown of *Cct2* in 661W cells decreased the levels of Gβ1 after 4 d of treatment (Fig. 5C). In combination, these results suggest that CCTβ and CCTγ have critical roles in the retina to maintain the physiology of the photoreceptor cells and the RGCs, and they also regulate its major target, Gβ1, an important molecule in the phototransduction pathway. Previously, it was shown that a deficit of Gβ1 in photoreceptor cells that was achieved by expressing a dominant negative form of PhLP evoked retinal degeneration in a mouse model by disturbing the function of transducin and the downstream phototransduction pathway^31^,^37^,^38^. Thus, it appears that reduced activity of the CCT machinery can contribute to the development of macular degeneration, as well as retinal dystrophy.

## Discussion

In the present study, the mutation of CCTβ is identified as a novel LCA-causative gene. Due to the clear diagnosis of LCA and the sufficient number of genome samples, the genetic analysis pointed out only one candidate gene with compound heterozygous inheritance. While it has to be noted that this *CCT2* gene mutation was found in sole consanguineous family and the following reports concerning the *CCT2* mutation with LCA will be anticipated. The CCT chaperone machinery found to be affected by the structurally instable T400P and R516H mutant proteins that exhibited an aberrant affinity to the CCTγ subunit adjacent to CCTβ. Moreover, the T400P- and R516H-carrying patient-derived T cells and iPSCs exhibited less proliferation. The T400P and R516H mutants also exhibited limited rescue effects on cell proliferation compared to wild-type CCTβ. Both subunits, CCTβ and CCTγ, were expressed in the cells in retinal ganglion cell layer and near the connecting cilium in the photoreceptor cells. In addition, levels of Gβ1, a major target of the CCT complex, and a critical protein in the phototransduction pathway, were decreased following the knockdown of *Cct2* in a mouse photoreceptor cell line.

According to recently developed functional gene ontology classifications, LCA-causative gene mutations have been divided into the following major groups: phototransduction (*GUCY2D, AIPL1*), retinoid cycling (*LRAT, RPE65, RDH12*), cellular maintenance (*AIPL1, TULP1, RD3*), ciliary function (*CEP290, IQCB1, LCA5, RPGRIP1, TULP1*), guanine synthesis (*IMPDH1*), and retinal development (*CRB1, OTX2, CRX*)^39^. In particular, aryl-hydrocarbon-interacting protein-like 1(*AIPL1)* is also a chaperone protein and it governs rod cGMP phosphodiesterase (PDE) biogenesis^40^. A defect in this molecular chaperone has been shown to indirectly contribute to LCA by affecting the phototransduction pathway. Growth differentiation factor 6 (GDF6), a growth factor that belongs to the transforming growth factor-β (TGF-β) family of proteins and controls retinal development, has also recently been reported to be a causative agent of LCA. Even though GDF6 is considered to be a developmental protein, mutated *GDF6* evokes LCA. Thus, it appears that a diverse set of conditions can lead to LCA, and mutation-based analyses of disease onset would be a great value in further understanding and establishing medical treatments for LCA.

It has recently been demonstrated that *CCT2* has roles in the folding of β-propeller proteins, such as Gβ1^41^, in addition to its major target proteins, actin and tubulin. In a study of the co-chaperone protein, PhLP, use of a dominant negative form lacking the N-terminal domain demonstrated that this region is necessary for the binding of nascent Gβ1. These results also showed the importance of Gβ1 in retinal homeostasis. In a transgenic mouse model expressing a dominant negative form of PhLP1 (dnPhLP^−tg^), deficits in Gβ1 in the retina are observed, and this is associated with severe retinal degeneration after birth due to transducin-associated phototransduction failure^31^,^42^. CCTβ has also been reported to interact with chaperonin type BBSs, including BBSs 6, 10, and 12, and lower levels of expression for a subset of BBSs has been observed in the dnPhLP^−tg^ retina^31^. Taken together, these results indicate that there may be a close link between chaperoning activity and cilium transport. On the other hand, the *CCT2* mutation-carrying LCA patients do not exhibit the clinical manifestations of BBS or ciliopathy in this study. This provably because the *CCT2* has more broad function in development by chaperoning the some critical developmental proteins while the BBS has more dominance in cilia-associating function. Though both CCTβ and CCTγ took a connecting cilium-like localization in photoreceptors, the association between CCT-BBS functional axis and human disease has to be elucidated in the future.

Artificial gene manipulation of other CCT family members, including *CCT3, CCT5,* and *CCT7*, have also been shown to evoke developmental eye defects in several zebrafish mutant lines (http://zebrafish.org/home/guide.php). In one of the well-characterized *CCT3* mutant zebrafish lines, *non tectal neuron (ntn*), a retinal phenotype was observed that involved fewer axonal projections of the RGCs to the lateral geniculate nucleus (LGN)^36^,^43^. The retinal ganglion cell deaths was observed in the *ntn* mutants probably because of lacking the trophic factor from LGN. It is possible that mutations in *CCT2* could lead to genetic retinal diseases based on this uncoordinated fashion of delayed development, in this case, the failure of axonal projection to the LGN. It is also a well-known fact that the migrating retinal astrocytes and the following vascular formation after birth closely associate with RGC axons^44^,^45^. Therefore, the axonal disruption of RGC could trigger the hypoplastice retinal development shown in the patients. It is an increasing fact to introduce the optic-cup and retinal differentiation in 3D or 2.5D organoid cultures from patient-derived iPSCs to evaluate the onsite genetic impacts. Recapitulate the phenotype in the optic organoid cultures will become a powerful analytic approach. Unfortunately, the patient-derived *CCT2* mutation-carrying iPSCs have a few disadvantages to introduce organoid cultures and was not applicable this time. Firstly, due to the significantly less proliferative activity of patient-derived *CCT2* mutation-carrying iPSCs, optimizing the total cultivation time, induction time point and endpoint validation time point to compare the phenotype of parental iPSCs were all difficult. Secondly, the *CCT2* phenotype in retina is probably associating with the RGC axon projection to LGN. It has been reported that the truncated mutation of CCTγ mutant zebrafish exhibited fewer axonal projections of the RGCs to the LGN and increased number apoptotic RGCs simultaneously^36^. This is probably because RGC-geniculate communication is required for further RGC survival and maturation. In order to validate these hypotheses, it is critical to utilize the *in vivo* phenotypic model that allows us to evaluate the orchestrate development of RGC with axon projection to the LGN. Recently, the genome editing by CRISPR-Cas9 is also applicable to iPSCs to validate the mutational impacts and rescue effect. This technique will provide the onsite impacts of *CCT2* mutation in retinal development and eliminate the trophic effect from LGN. Combining all these techniques and further analyses will be important to clarify the *CCT2* function in detail. The forward genetics in experimental animals, in combination with genome wide sequencing in human patients, has the potential to elucidate the causes of various developmental diseases.

Gene mutations in rat *CCT4* (C450Y of CCTδ)^46^,^47^ and human *CCT5* (H147R of CCTε)^48^ have been reported to cause sensory neuropathy, although the underlying mechanisms of disease onset have not been elucidated. Recently, the effects of these two mutations on the CCT machinery were analyzed using *in vitro* expression of mutant homo oligomer models^49^,^50^. Both of the gene mutations evoked sensory neuropathy, albeit via two distinct mechanisms. Mutant C450Y of CCTδ was found to be structurally instable and it failed to form the CCT ring complex. In contrast, mutant H147R of CCTε exhibited normal CCT ring complex formation, yet the activity of the CCT machinery was reduced^49^,^50^. This analysis of the biochemical features of these eukaryotic mutant CCT subunits in a homo oligomer model established in prokaryotic host cells has the disadvantage that CCT is a eukaryote-specific chaperone and is not functional in prokaryotes. Thus, it is difficult to further investigate the physiology of the hetero-oligomer complex in this model. On the other hand, in the present study, the patient iPSCs demonstrated that maintenance of CCT machinery affects cell proliferation. It is noteworthy that the mutations, T400P and R516H, in CCT2 were not embryonic lethal, but resulted in a retinal phenotype. Taken together, these results suggest that there is a distinct threshold for the impact of mutations on subunit-target interactions in specific cell types as well as the tissue specificity. Thus, disease manifestation due to CCT mutations may appear in cells/tissues where CCT subunit-governed targets have greater importance in maintaining cellular physiology. By characterizing the chemistry of the respective CCT subunits and identifying the specific targets of the CCT complex, it may be possible to further elucidate which target proteins are crucial for specific cell types, thereby providing novel insights into our understanding of retinal development and the associated pathoetiology of hereditary retinal diseases.

In conclusion, two novel compound heterozygous mutations in *CCT2* were identified in association with a family affected by LCA. These mutations affected CCTβ-associated chaperone activity which has an essential role in retinal development and photoreceptor physiology.

## Methods

### Whole Exome Analysis

For the human sample experiments, informed consents for all individuals were obtained in written format from the parents. DNA samples from all of the family members were subjected to WES to identify the responsible gene mutations (Fig. 1A). Libraries for WES were prepared from the DNA samples using an exon capture kit (SureSelct ver. 4+ UTR, Agilent Technologies, Santa Clara, CA), according to the manufacturer’s instructions. The exons were sequenced as 100 basepair paired-end reads by an Illumina HiSeq2000 (Illumina, San Diego, CA). The sequence data obtained were then analyzed to extract potentially causal mutations. Briefly, sequences of the whole exome were compared with a reference human genome (hs37d5), and 5,144,654 mutations were identified. Only mutations that resulted in a change in amino acid sequence were selected as candidate mutations (n = 681), the remaining common mutations were excluded. Using a pattern of inheritance with the parents, one candidate causal gene was identified (Supplemental Table 1). In the RetNet database, only a gene mutation present in *chaperonin-containing TCP-1, subunit 2 (CCT2*) was registered as a loci for inherited retinal diseases and was speculated to be a disease-causing mutation.

### Three-Dimensional Mutant Protein Structure Prediction

To predict whether these mutations in CCTβ directly impact disease onset, molecular modeling of the hetero-octamer CCT machinery was conducted using a molecular visualization, modeling, and dynamics program, YASARA (www.yasara.com). Amino acid sequences of the eight CCTα-θ subunits were retrieved from the UniProtKB database (http://www.uniprot.org/uniprot), and the atomic structures for each domain were individually modeled by homology. The final structure of the hetero-octamer was built using an extensible molecular modeling program, UCSF Chimera (https://www.cgl.ucsf.edu/chimera/), with the Cα-atom structure of bovine CCT used as a structural template (PDB file: 3iyg). The CCTβ missense mutations, T400P and R516H, were generated and positioned in an optimized conformation. Full atomic structures of the wild type and mutant octamers were minimized iteratively to achieve a global energy minimum structure. Finally, octamer stereochemistry was optimized and equilibrated using 1 nanosecond molecular dynamics in water.

### Establishment of LCA Patient-Specific iPSCs

Prior to obtaining iPSCs from blood samples, the patients were fully informed and all procedures performed were approved by the ethics committee of the National Hospital Organization Tokyo Medical Center. All the experiments were carried out in accordance with the approved guidelines. Circulating T-cells in the blood samples were obtained by centrifugation with Ficoll reagent, and the Yamanaka 4 factors were induced by Sendai-virus infection. After transforming the iPSCs, alkaline phosphatase activity was assayed, and then the undifferentiated stem cell state was confirmed by detecting expression of Nanog, OCT3/4, TRA81, and SSEA4.

### Plasmids and Biochemical Studies

The cDNA clone of human *CCT2* was obtained from NITE Biological Resource Center (NBRC). The cDNA was subcloned into the BamHI site of pEF-BOS-FLAG vector^51^ and the mutations responsible for T400P and R516H were introduced by KOD Mutagenesis Kit (TOYOBO) respectively. The human CCTβ, T400P and R516H were transfected into HEK293T cells^34^ with TransIt Pro Transfection Kit (Mirus) according to its manufacturer’s instructions. A mini-PROTEAN TGX Gel and Transblot Turbo system (BioRad) were used for SDS-PAGE Western blotting (WB) according to the manufacturer’s instructions^34^. Actin detection was used as a loading control and for the normalization of CCTβ levels. Detected bands against CCTβ or actin were quantitated using ChemiDoc XRS+ with the Image lab software package (BioRad). Intracellular protein stability was monitored by treating transfected cells with proteasomal degradation inhibitor, MG132, and protein synthesis was abolished with cychloheximide (CHX) during 3 h. Immunoprecipitates (IPs) by FLAG-tag were prepared with M2-FLAG sepharose (Sigma) according to its manufacturer’s protocol^34^. The IPs of wild-type, T400P, and R516H forms of CCTβ were next subjected to SDS-PAGE and then were silver stained (Silver Quest). Then the T400P specific bands were in-gel digested and processed for LC/MS/MS as previously reported^52^. C57BL/6J retina was carefully removed from the posterior part of the eye and the retinal pigment epithelium (RPE) was then scraped from the remained sclera tissue to assess the expression of CCTβ in the mouse eye. The animal experiment was carried out in accordance with the Guide for the Care and Use of Laboratory Animals (National Institutes of Health) and the Association for Research in Vision and Ophthalmology Statement for the Use of Animals in Vision Research and approved by the Tokyo Medical Center Experimental Animal Committee. The tissue lysate was analyzed by WB using mini-PROTEAN TGX Gel and TransBlot Turbo system (BioRad) as mentioned above.

### Proliferation Assay

Cell proliferation was monitored by MTS Assays (Cell Titer, Promega). The proliferation of iPSCs that were seeded onto feeder-coated 96-well plates (1 × 10^3^ cells/well) was monitored for 3 days with MTS assays. Growth rates according to spheroid size for iPSCs grown in serum-free embryoid body cultures were described previously^53^. Two-sample *t*-test was performed according to the variance results respectively. *P* values less than 0.05 were considered to be statistically significant.

### SiRNA and Stable Expression by Lenti Virus

The siRNA system (Sealth RNAi siRNA, Life Technologies) for Mouse *CCT2* and a control siRNA were used with RNA iMAX reagents (Life Technologies) to knockdown expression of endogenous mouse *Cct2* in 661W cells, a mouse cone photoreceptor cell line. Transfection of a siRNA specific for mouse *Cct2* that did not exhibit off-target effects on human *CCTβ* in HEK293T cell line was performed and no off-target effect was confirmed by WB (data not shown). The human wild type CCT*β*, T400P and R516H stably expressing 661W cells were established by lenti virus system with CSII-CMV-MCS-IRES2-Bsd vector and the packaging plasmids (RIKEN BRC).

### Immunohistochemistry

Eyes from 5-week-old male C57B/6j mice were dissected and fixed in 4% paraformaldehyde. After sucrose replacement, the lens was removed from each eye and left retinal tissues were routinely processed to obtain 14 μm-thick cryosections. The following antibodies were used for staining: anti-CCTβ (Cell Signaling Technology), anti-CCTγ (Abcam), and anti-Gβ1 (Abcam). Briefly, the cryosections were permeabilized with 0.4% Triton X-100 in phosphate-buffered saline (PBS) for 15 min. For negative controls, a mixture of rabbit IgGs (Dako) was used instead of primary antibody. The specimens were counterstained with Hoechst33642. The LSM700 and Zen Systems (Zeiss) were used for confocal microscopy.

## Additional Information

**Accession codes:** Submitted to NCBI ClinVar database. http://www.ncbi.nlm.nih.gov/projects/SNP/handle.cgi?sid=IWATA-LAB_1423973816.

**How to cite this article**: Minegishi, Y. *et al.* *CCT2* Mutations Evoke Leber Congenital Amaurosis due to Chaperone Complex Instability. *Sci. Rep.* **6**, 33742; doi: 10.1038/srep33742 (2016).

## Supplementary Material

Supplementary Information
