# Supplementary material for: CCT2 Mutations Evoke Leber Congenital Amaurosis due to Chaperone Complex Instability
Source: Sci Rep. 2016 Sep 20;6:33742. doi: 10.1038/srep33742 (PMC5028737; doi:10.1038/srep33742)
Supplement: Supplementary Information [file srep33742-s1.doc]

***CCT2* Mutations Evoke Leber Congenital Amaurosis due to**

**Chaperone Complex Instability**

Yuriko Minegishi ^1, 7^, XunLun Sheng ^2, 7^, Kazutoshi Yoshitake ^3^, Yuri Sergeev ^4^, Daisuke Iejima ^1^, Yoshio Shibagaki ^5^, Norikazu Monma ^3^, Kazuho Ikeo ^3^, Masaaki Furuno ^6^, Wenjun Zhuang ^2^, Yani Liu ^2^, Weining Rong ^2^, Seisuke Hattori ^5^, Takeshi Iwata ^1, *^

^1^ Division of Molecular and Cellular Biology, National Institute of Sensory Organs, National Hospital Organization Tokyo Medical Center, Tokyo, Japan. ^2^ Ningxia Eye Hospital, Ningxia People’s Hospital, Ningxia, China. ^3^ Laboratory of DNA Data Analysis, National Institute of Genetics, Shizuoka, Japan. ^4^ National Eye Institute, National Institutes of Health, Bethesda, MD, USA. ^5^ Division of Biochemistry, School of Pharmaceutical Science, Kitasato University, Tokyo, Japan. ^6^ RIKEN Center for Life Science Technologies, Division of Genomic Technologies, Life Science Accelerator Technology Group, Transcriptome Technology Team, Yokohama, Japan.

**Supplementary Figure S1**

**
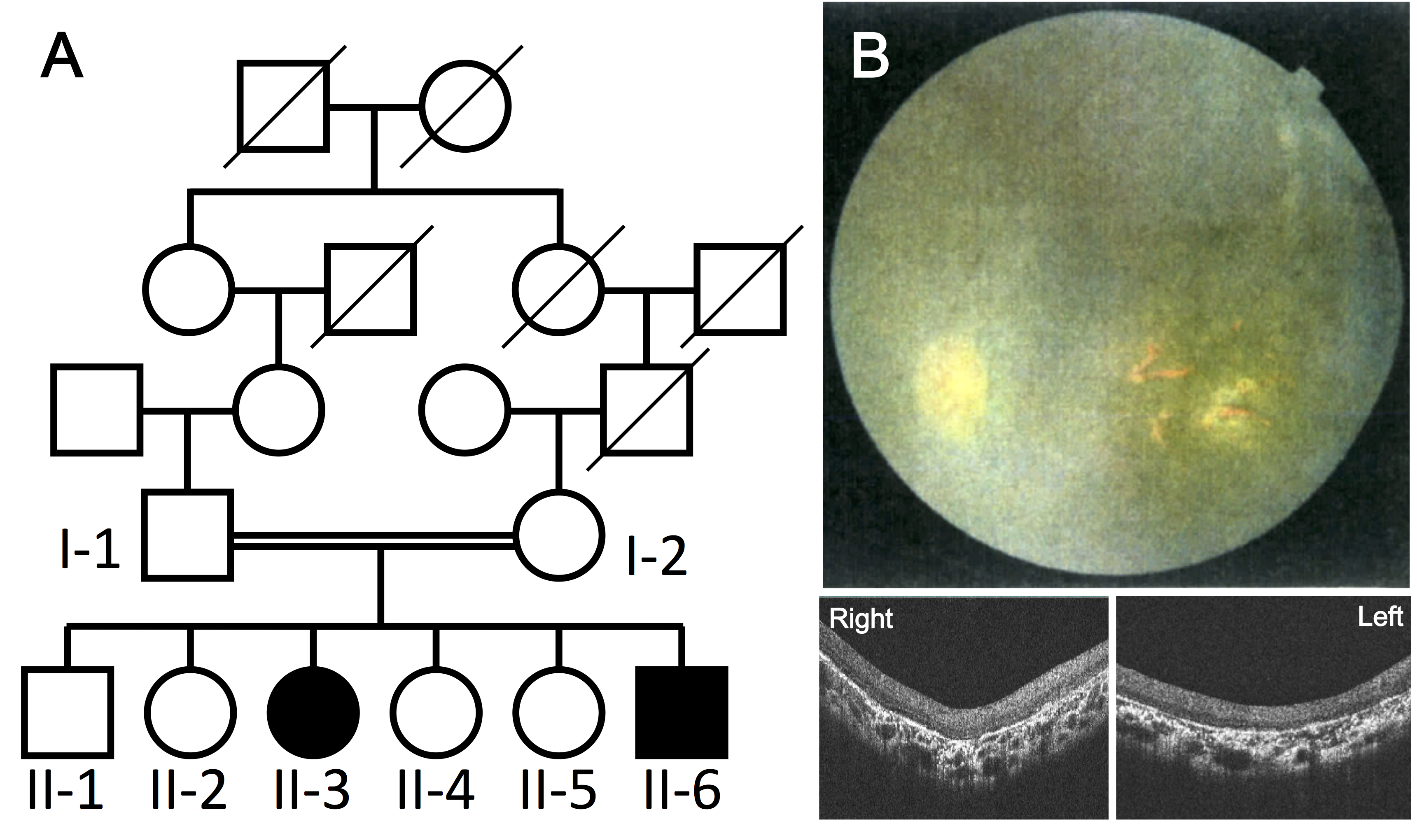
**

**Family tree and clinical observations**

**(A)** The parents of the patients examined are second cousins. **(B)** Clinical features of individual, II-6. Upper panel: Color fundus photographs that show severe dystrophy and macular degeneration. A fundus image of the right eye was not available. Lower panel: An OCT examination revealed severe macular degeneration and a hypoplastic structure for the retina.

**Supplementary Table S1**

**
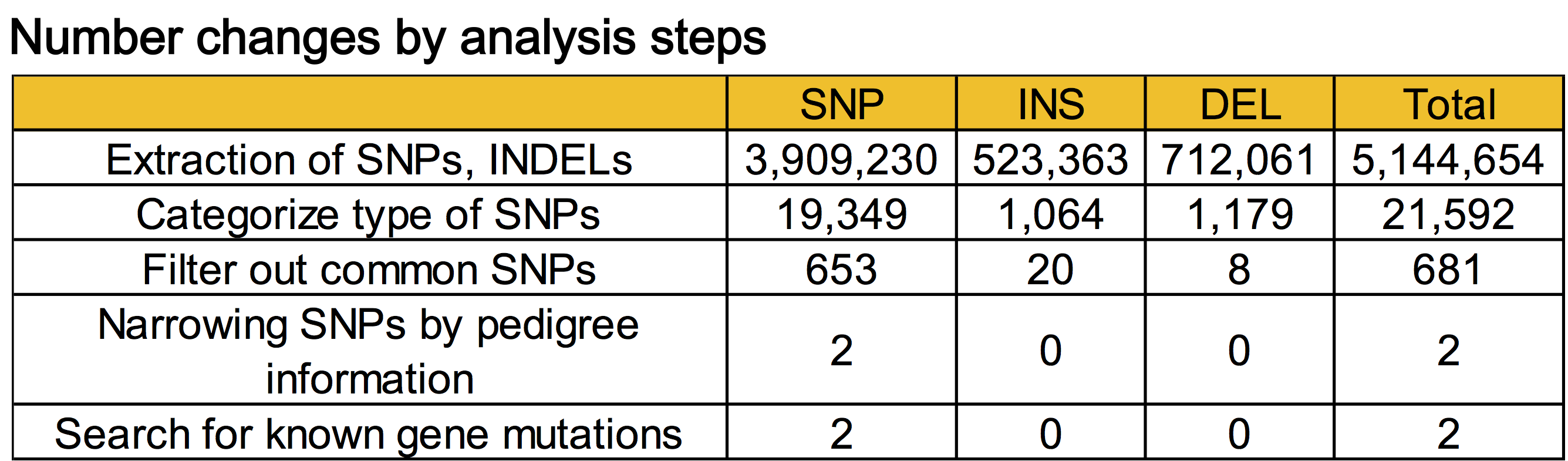
**

**Scheme and summary of the whole exome sequencing analysis that was performed.**
